# The Effect of Telerehabilitation on the Outcome of Arthroscopic Rotator Cuff Repair: A Meta-analysis of Randomized Clinical Trials

**DOI:** 10.1016/j.arrct.2026.100635

**Published:** 2026-05-13

**Authors:** Wu Jiao, Han Chang-Xu, Li Li, Li De-Long, Zhuang Shu-Yuan, Dong Si-Hong, Dang Yu, Pan Linlin

**Affiliations:** a School of Nursing, Inner Mongolia Medical University, Hohhot, China.; b Department of Rehabilitation, The Second Affiliated Hospital of Inner Mongolia Medical University, Hohhot, China.; c Department of Nursing, The Second Affiliated Hospital of Inner Mongolia, Hohhot, China.

**Keywords:** Meta-analysis, Randomized clinical trials, Rehabilitation, Rotator cuff-related diseases, Shoulder function, Telemedicine

## Abstract

**Objective:**

To evaluate the effectiveness of telerehabilitation in the postoperative treatment of rotator cuff diseases following arthroscopic repair.

**Data Sources:**

Randomized controlled trials comparing the effects of telemedicine and conventional treatment on patients after arthroscopic rotator cuff repair were retrieved from PubMed, Embase, Cochrane Library, Web of Science, and other databases up to December 2024.

**Study Selection:**

The Cochrane risk of bias tool was used for quality assessment. Meta-analysis was conducted using RevMan 5.4 software. Forest plots and Egger’s test were used to estimate publication bias. The results were integrated using the pooled integration method.

**Date Extraction:**

Two researchers independently conducted the data extraction. Discrepancies were resolved through discussion.

**Data Synthesis:**

A total of 5 studies were included, involving 317 participants (telerehabilitation group, 157; conventional group, 160), with a mean age ranging from 26.5 to 63.9 years and a gender distribution of 74 males and 243 females. Follow-up periods ranged from 12 to 52 weeks. After treatment, functional outcomes measured by the Constant-Murley score in the telerehabilitation group were significantly better than those in the conventional treatment group. Compared with conventional treatment, telerehabilitation significantly improved upper limb function evaluated by the quick disability of the arm, shoulder, and hand score and shoulder strength, and improved shoulder function during the posttreatment period and at the final follow-up.

**Conclusion:**

The combination of home monitoring devices with traditional rehabilitation methods is receiving significant attention. It offers a broader range of high-quality care services and is expected to reduce medical costs associated with face-to-face treatment.

Arthroscopic rotator cuff repair is a preferred surgical choice due to its minimally invasive nature and high level of patient satisfaction.^1^ However, shoulder surgeries are associated with various complications, such as scar formation, adhesions, pain, and stiffness.^2^ Previous research and practice guidelines have emphasized the significance of postsurgical rehabilitation plans in achieving positive outcomes for patients.^3^^,^^4^ An effective rehabilitation plan can address the key issues related to rotator cuff function, pain, and muscle strength.^5^

Telemedicine involves the use of communication technologies and electronic information for remote diagnosis, treatment, and management. It has become increasingly common in orthopedic surgery, including initial consultations, perioperative care, and rehabilitation.^6^ There is evidence suggesting that telemedicine can effectively alleviate early shoulder pain and stiffness following shoulder surgery and improve rehabilitation outcomes.^7^ Furthermore, telemedicine can address geographical and social barriers that patients may encounter when accessing healthcare.^8^ Although telemedicine is gaining recognition, there is a lack of meta-analyses determining its effectiveness specifically for patients undergoing arthroscopic rotator cuff repair. This study aims to evaluate the effectiveness of telemedicine in the postoperative follow-up period for these patients undergoing arthroscopic rotator cuff repair and provide clinical references for their rehabilitation management.

## Methods

### Search strategy

**Fig 1:**
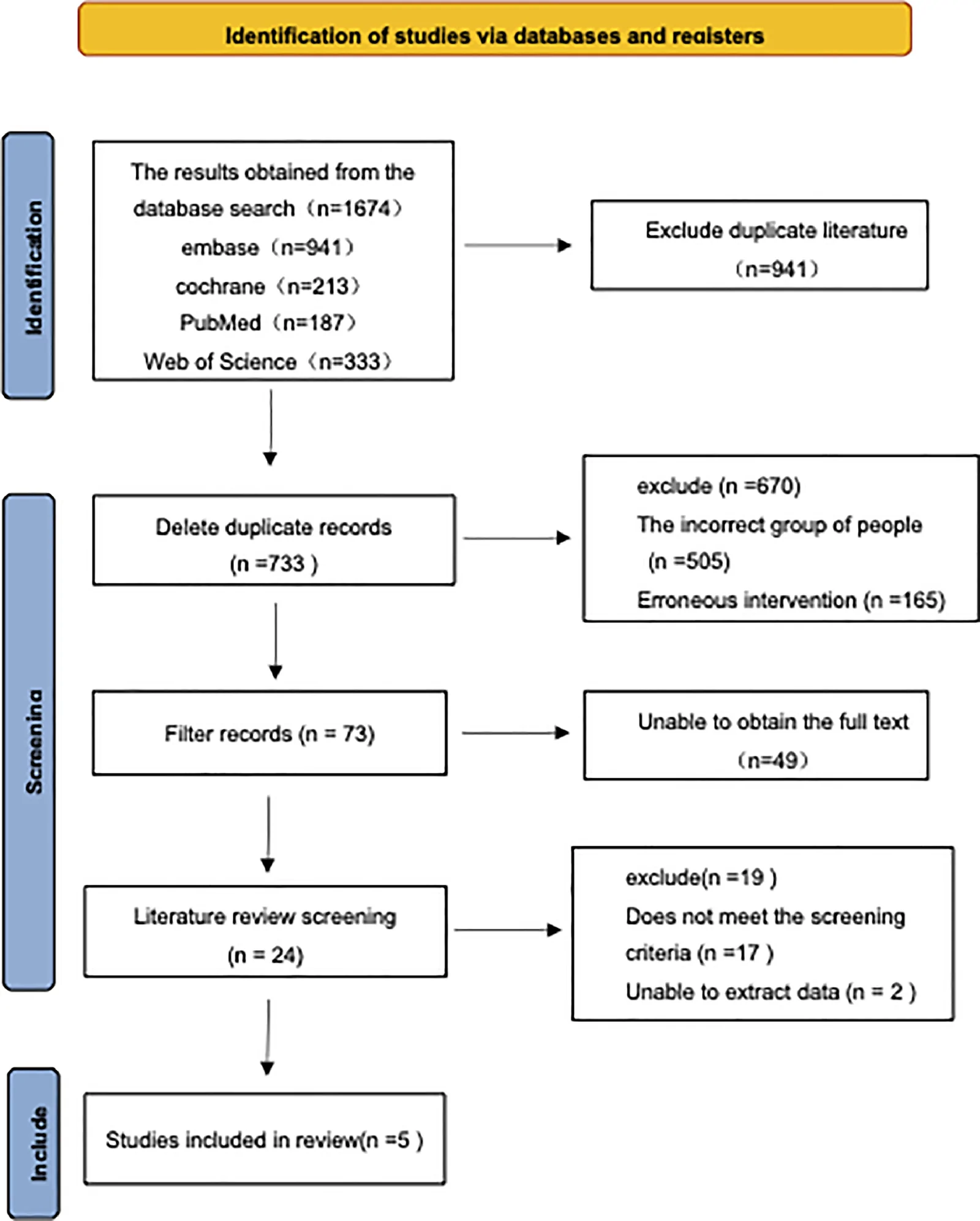
Flowchart of research screening process.

This study follows the PRISMA guidelines (see supplemental table S1).^9^ The study protocol was registered in PROSPERO (http://www.crd.york.ac.uk/prospero, registration no.: CRD420251018069). The search strategy was designed by 2 researchers using a combination of medical subject headings terms (remote rehabilitation, remote medical care, shoulder joint, joint replacement surgery, arthroscopy, and rotator cuff diseases) and free words. Data were retrieved from database inception to December 2024, including PubMed, Cochrane Library, Embase, and Web of Science. The flowchart of the research screening process is shown in figure 1. The detailed search strategy for PubMed is presented in supplemental table S2.

### Inclusion and exclusion

To determine the effectiveness of telemedicine technology for patients with rotator cuff injuries, this study was conducted according to the Participants, Intervention, Comparison, Outcome, and Study design (PICOS) framework. The guideline sets the qualification criteria for screening high-quality randomized controlled trials (RCTs) as follows^10^: (1) Participant: (a) age >18 years; (b) diagnosed with a rotator cuff tear and underwent arthroscopic rotator cuff repair. (2) Interventions: patients in the intervention group received any form of remote medical treatment, such as gamified supervised physical therapy, remote medical programs, digital therapy, telephone assistance programs, and video conferences. (3) Comparisons: patients in the control group received standard treatment or conventional rehabilitation therapy. Detailed conventional treatments are expressed in the search results and study characteristics. (4) Outcomes: the main outcomes were the Constant-Murley score (CMS), the Quick Disability Assessment Score for the upper extremity (quick disability of the arm, shoulder, and hand [QuickDASH]), range of motion (ROM), and pain scores. The abovementioned scores mainly cover indicators such as the ROM of the shoulder joint. (5) Study type:RCTs or studies investigated the impact of telemedicine on the shoulder joint function or pain of patients with rotator cuff injury (even if they were not RCTs, the baseline characteristics between the telemedicine group and the control group were similar). (6) Studies were excluded for the following reasons: (a) Did not use telemedicine technology to guide the recovery process; (b) Outcome indicators could not be quantified; (c) were comments, letters, meeting summaries, or systematic reviews; (d) were non-English; (e) full text could not be obtained.

### Data extraction

Two researchers independently conducted the data extraction. During the data extraction process, any discrepancies were resolved through discussion and negotiation. After including the literature, the following information was extracted: first author, publication year, publication country, surgical method, specific intervention measures, sample size, gender (male/female), age, baseline values, outcome indicators during the posttreatment and at final follow-up.

### Quality assessment

The risk of bias of the included RCTs was independently assessed by 2 researchers using the Cochrane risk of bias tool (ROB2).^a^ Each study was evaluated in 5 specific areas: randomization, deviation from the established intervention measures, missing outcome data, outcome indicators, and selective reporting of results. Each domain was independently rated by 2 researchers. The risk of deviation in each domain was classified as “low (green),” “with some concerns (yellow),” or “high (red).” Disagreements during the scoring process were resolved through discussions or by seeking advice from a third researcher. The assessment results are presented in the form of an offset risk map.

### Statistical analysis

The data collected from the included studies were analyzed using RevMan, version 5.4.^b^ Continuous data are presented as the mean value and SD.^11^ The heterogeneity among the studies was assessed using I². An I² value ≥50% indicated the presence of significant heterogeneity. Therefore, the random-effects model (where differences in distribution can affect the overall true effect) was used.^12^ The sources of heterogeneity were determined through sensitivity analysis (the elimination method one by one). Furthermore, subgroup analyses were conducted based on gender, age, cause of illness, and study location. On the contrary, an I² value <50% indicated a lower level of heterogeneity.^12^ Publication bias was evaluated using funnel plots and the Egger test.^13^ A 2-tailed *P* value <.05 indicated statistical significance.

## Results

### Retrieval results and study characteristics

Initially, 1674 records were retrieved. A total of 1669 studies were excluded because they did not meet the inclusion criteria. A total of 5 studies were finally included (157 in the telemedicine group and 160 in the control group). Table 1 summarizes the research characteristics and evaluation results. Among the 5 included studies,^14^, ^15^, ^16^, ^17^, ^18^ most were conducted in Europe: the United Kingdom (n=1),^16^ Spain (n=2),^17^^,^^18^ and Portugal (n=1),^15^ another study was conducted in South Korea (n=1).^15^

**Table 1:** Basic Characteristics of Included Literature

Study	Year	Country	Sample size (n)		Age, Mean (SD)			Sex (M/F)		Treatment Details		Treatment Duration	Follow-Up Duration	Surgical Procedures	Outcome
			I	C	I	C		I	C	I	C				
Shim et al^14^	2023	Korea	58	57	63.90 (6.2)	63.70 (6.7)		24/34	21/36	Rehabilitation program software, 3D depth camera, video call	Family exercise routine	12 wk	24 wk	Acromion reshaping surgery or labrum repair surgery	QuickDASH
Correia et al^15^	2022	Portugal	27	23	61.30 (7.0)	60.04 (6.8)		7/20	4/19	Inertial motion tracker	Traditional family rehabilitation	12 wk	12 mo	Arthroscopic rotator cuff repair surgery	QuickDASH, CMS, Strength
Marley et al^16^	2022	England	31	33	52.90 (19)	54.40 (8.5)		13/18	13/20	Use the application to provide exercise information and guidance to patients	Traditional home-based physical therapy	12 wk	12 wk	Arthroscopic shoulder surgery	QuickDASH
Pastora-Bernal et al^17^	2017	Spain	8	10	49.63 (10.08)	54.80 (11.84)		4/4	6/4	Send the exercise videos and pictures generated by the therapist via email	Face-to-face traditional physical therapy	12 wk	12 wk	Arthroscopic acromioplasty	CMS
Martine-Rico et al^18^	2018	Spain	36	34	26.50 (5.5)	29.0 (9.75)		26/10	28/6	Telephone-assisted care program	Traditional physical therapy	12 mo	12 mo	Arthroscopic Bankart repair	QuickDASH

Abbreviation: C, control; I, Intervention.

During the treatment and follow-up period, the intervention group received ≥12 weeks of telerehabilitation (eg, remote programs, telephone assistance, and video conferences), whereas the control group received traditional rehabilitation.

The telemedicine team employed various tools or methods, such as 3D camera sensors (which can convert the geometric information of the scene into depth information and track the movements of 25 joints of the upper and lower limbs)^14^; inertial motion tracker (providing real-time audio and video biofeedback during the movement process)^14^^,^^15^; the physical therapy through sports games is tailored to the patient’s abilities based on the duration and difficulty of the games (the patient’s abilities are determined by their physical therapist)^16^^,^^18^; the interaction with the physical therapist involves the therapist providing images, videos and parameters of each exercise plan via email for treatment purposes, as well as conducting telephone follow-ups. The therapist also conducts regular telephone follow-ups to ask and answer relevant questions.^17^ The methods adopted by the control group mainly included family-based exercise routines,^14^^,^^15^ conventional face-to-face physical therapy,^16^^,^^17^ and conventional treatment.^18^ Specifically, the family-based exercise routines consisted of a structured set of home exercises prescribed by a physical therapist, typically including range-of-motion exercises, stretching, and progressive strengthening of the rotator cuff and scapular stabilizers. Patients were provided with written instructions or illustrated booklets and were instructed to perform the exercises daily for 12 weeks, with no remote monitoring or real-time feedback. All patients suffered from arthroscopic rotator cuff repair for various reasons, such as shoulder cuff injuries,^15^^,^^19^ shoulder dislocation,^18^ and subacromial impingement syndrome.^16^^,^^17^ All the included studies reported the corresponding preoperative and postoperative shoulder scores through relevant outcome indicators, including the QuickDASH score,^14^^,^^15^^,^^18^ the CMS,^15^^,^^17^ and Strength score.^15^^,^^17^

### Quality assessment

**Fig 2:**
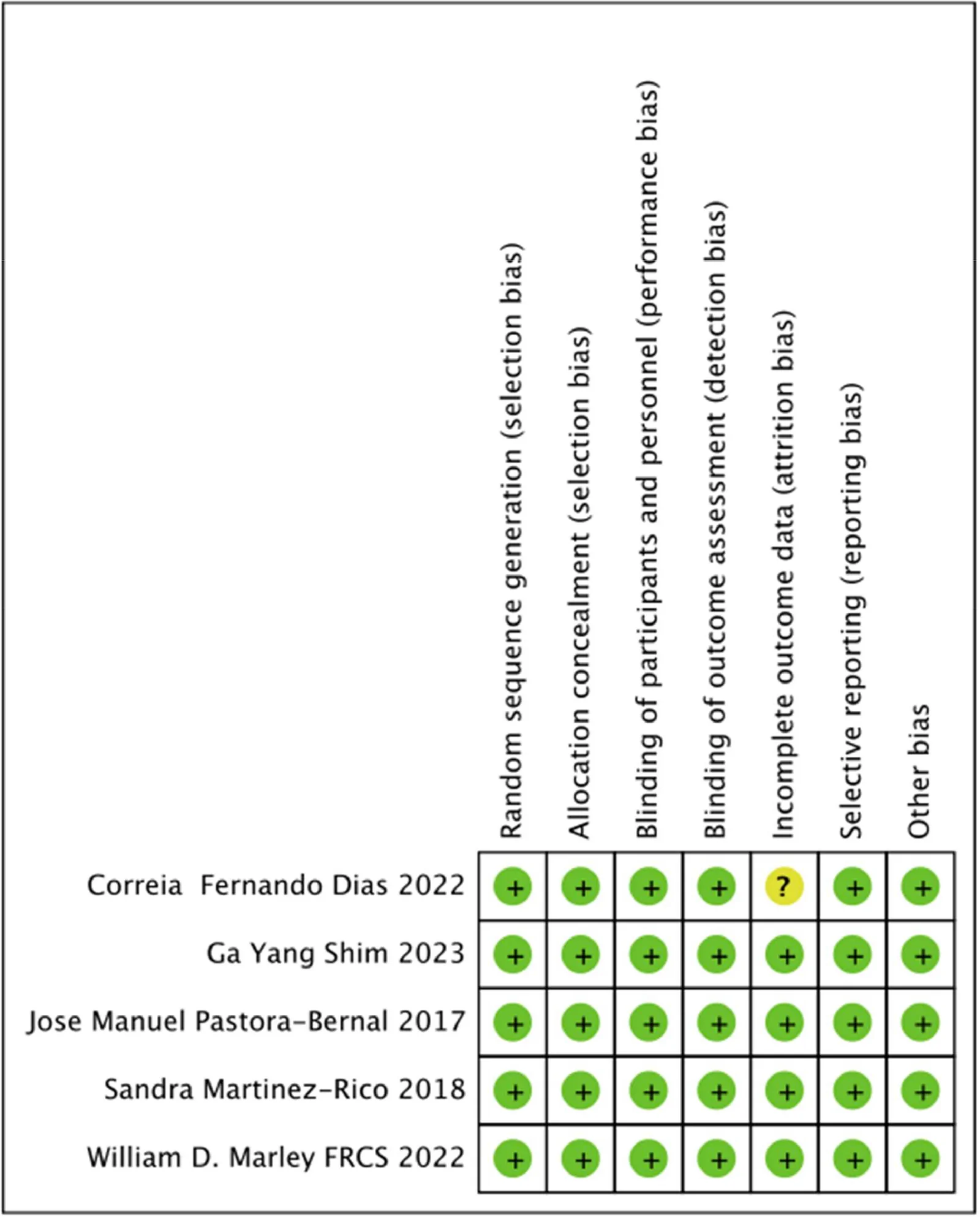
Results of research quality evaluation.

Most studies employed random sequences, reported results without selectivity, and determined the follow-up duration and outcomes for both groups. Most studies achieved allocation concealment and blinding of participants and researchers. The quality assessment results are shown in figure 2.

### Results of meta-analyses

#### Shoulder function measured by the CMS

**Fig 3:**
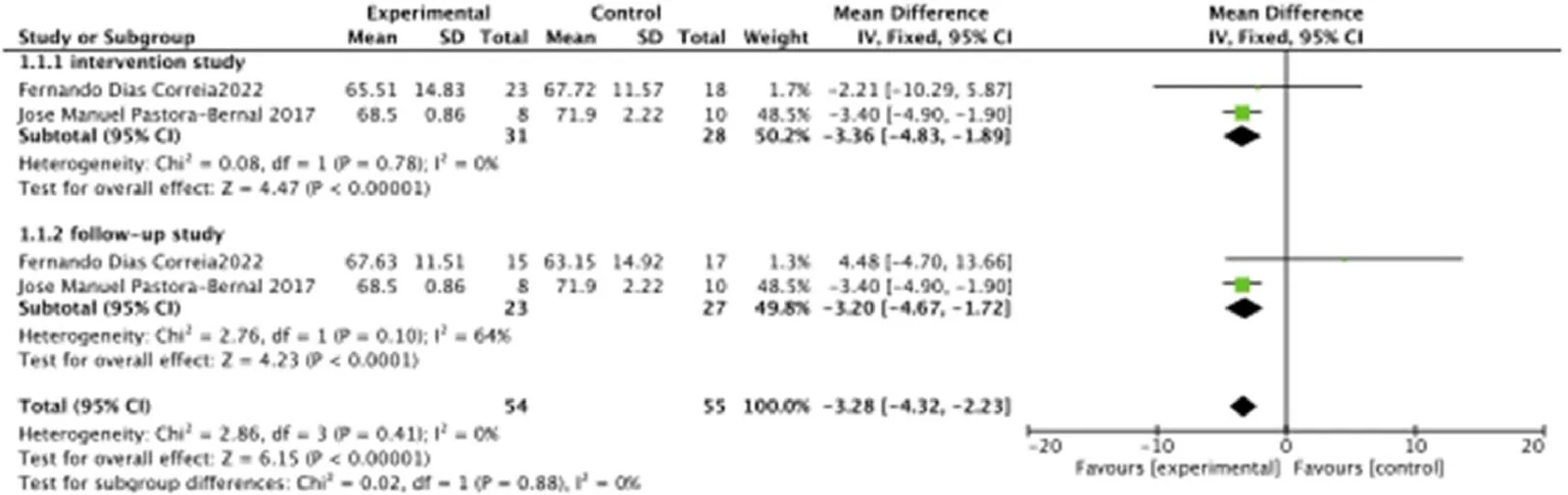
Forest plot of the impact of telemedicine on the Constant-Murley score (CMS).

The CMS is an effective tool for assessing the functional impairment and recovery of the shoulder joint in patients with rotator cuff disorders.^20^ The available evidence indicates that the CMS can assess shoulder joint pathology across 4 aspects,^21^ it includes 2 subjective aspects (pain and daily life activities) and 2 objective aspects (joint movement and strength). Two studies reported the CMS data.^15^^,^^17^ After treatment, 59 participants were involved (31 in the intervention group and 28 in the control group), and 50 participants were followed up (23 in the intervention group and 27 in the control group). The meta-analysis showed low heterogeneity among the various items (I^2^=0%; *P*=.41). The combined effect size using a random-effects model indicated that the intervention group had higher scores than the control group (mean difference [MD]=−3.28; 95% confidence interval [CI], −4.32 to −2.23; *P*<.0001). After the treatment, the analysis showed that compared with the control group, telemedicine significantly improved the ROM of the shoulder joint (MD=−3.36; 95% CI, −4.83 to −1.89; *P*<.0001), as shown in figure 3. Follow-up evaluations were conducted for both studies (duration of 12 weeks each^21^^,^^22^). Therefore, we conducted a meta-analysis to examine the medium- and long-term effects of telemedicine on shoulder function. The results showed that compared with the control group, the patients in the telemedicine group had significant changes in their shoulder mobility, but the improvement effect did not reach statistical significance (MD=–3.20; 95% CI, –4.67 to –1.72; *P*<.0001). There was significant heterogeneity in the final follow-up (I^2^=64%), so a sensitivity analysis was conducted to determine the source of heterogeneity. The analysis results were relatively stable, and Egger’s test showed no obvious publication bias (*P*=.217; since the number of final follow-up evaluations included was limited, Egger’s test was not performed).

#### QuickDASH assessment of upper limb function

**Fig 4:**
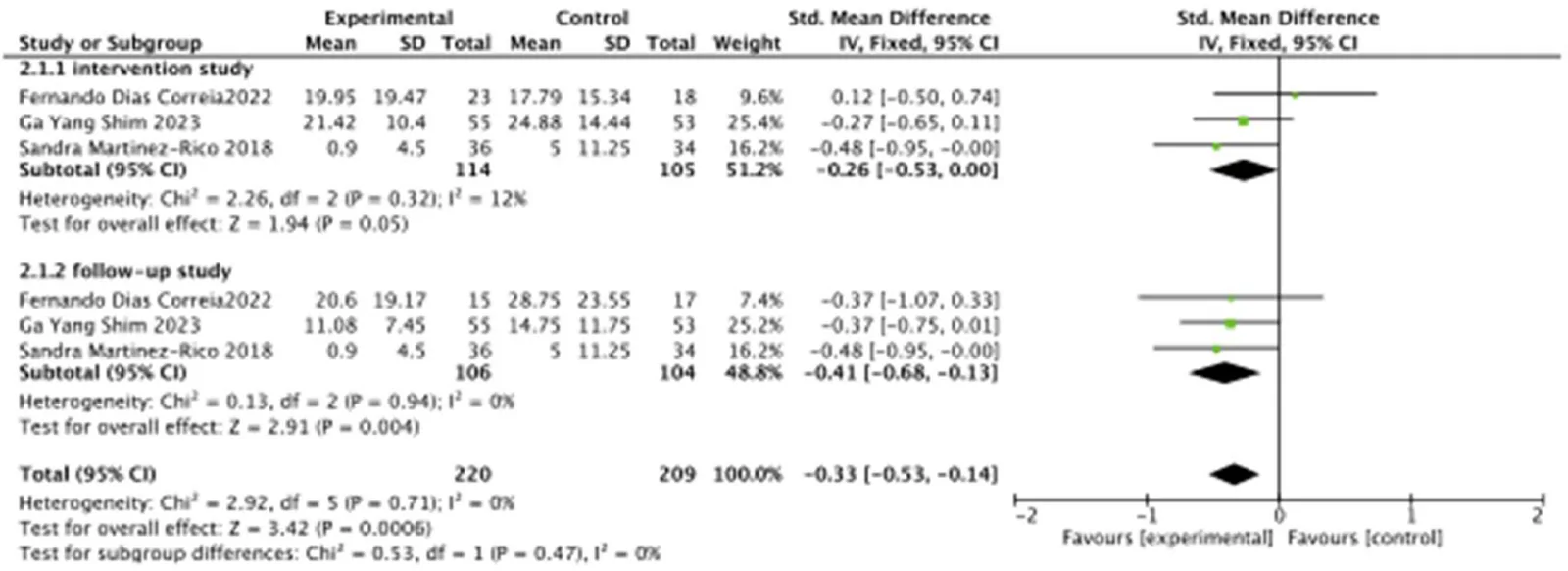
Forest plot showing the impact of telemedicine on QuickDASH.

The QuickDASH score questionnaire is a widely used standardized tool for assessing the upper limb function and symptoms of patients, including pain (at rest, during activity, and during sleep), muscle strength, and stiffness. The lower the patient’s score is, the better the function of the upper limbs will be.^23^ Three studies reported QuickDASH as an outcome,^14^^,^^15^^,^^18^ A total of 219 participants (114 in the intervention group and 105 in the control group) were involved in the treatment, and 210 participants (106 in the intervention group and 104 in the control group) were followed up. The meta-analysis showed low heterogeneity among the various items (I^2^=0%; *P*=.71). The combined effect size calculated using a random-effects model indicated that the intervention group had higher scores than the control group (MD=−0.33; 95% CI, −0.53 to −0.14; *P*=.0006). After the treatment, the meta-analysis showed that compared with the control group, telemedicine significantly improved upper limb function and symptoms (MD=−0.26; 95% CI, −0.53 to 0.00; *P*=.05), as shown in figure 4. In these 3 studies, follow-up evaluations were conducted (with 2 of the studies having a follow-up period of 12 weeks,^14^^,^^15^ another study had a follow-up period of 12 months,^18^ 106 cases in the intervention group and 104 cases in the control group). We conducted a meta-analysis on the medium- and long-term effects of telemedicine on shoulder function. The results showed that compared with the control group, patients in the telemedicine group significantly improved upper limb function and symptoms (MD=−0.41; 95% CI, −0.68 to −0.13; *P*=.004).

#### Strength score assessment of shoulder strength

**Fig 5:**
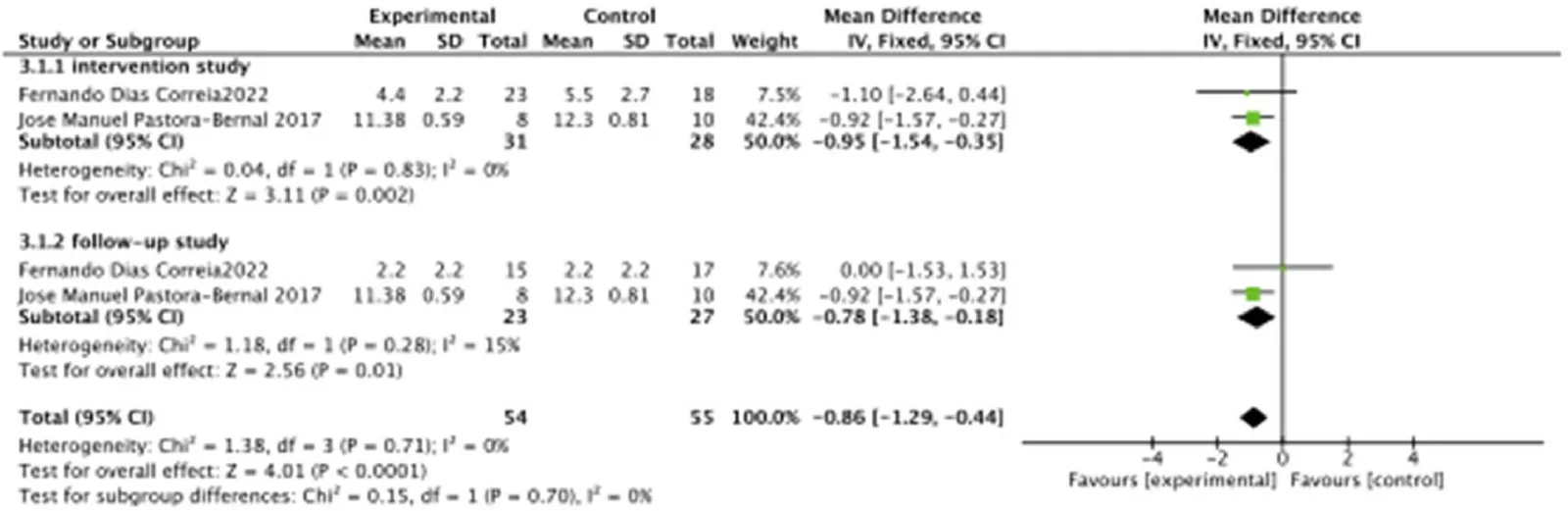
Forest plot of the impact of telemedicine on Strength.

The strength score is widely used in the diagnosis of shoulder joint diseases, the evaluation of treatment effects, and rehabilitation guidance. The higher the Strength score, the better.^22^ In the assessment of shoulder joint function, “Strength” refers to the strength of the shoulder muscles.^22^ Two studies used “Strength” as an outcome.^15^^,^^17^ A total of 59 participants (31 in the intervention group and 28 in the control group) were involved in the treatment, and 50 participants (23 in the intervention group and 27 in the control group) were followed up. The meta-analysis showed low heterogeneity among the various items (I^2^=0%; *P*=.71). The combined effect size using a random-effects model indicated that the intervention group had higher scores than the control group (MD=−0.86; 95% CI, −1.29 to −0.44; *P*<.0001). After the treatment, the meta-analysis showed that compared with the control group, telemedicine significantly improved the strength of the shoulder muscles (MD=–0.95; 95% CI, −1.54 to −0.35; *P*=.002), as shown in figure 5. In these 2 studies, follow-up evaluations were conducted (with the follow-up period being 12 weeks for both^15^^,^^17^). Therefore, we conducted a meta-analysis to examine the medium- and long-term effects of telemedicine on shoulder function. The results showed that compared with the control group, patients in the telemedicine group significantly improved shoulder muscles (MD=−0.78; 95% CI, −1.38 to −0.18; *P*=.01).

## Discussion

To our knowledge, this is the first meta-analysis to assess the effectiveness of telerehabilitation for patients following arthroscopic rotator cuff repair. The results show that compared with traditional treatment, telerehabilitation significantly improves shoulder function, as measured by the CMS, the Quick Disability Assessment Score for the upper extremity (QuickDASH), and strength scores.

This study’s analysis indicates that telerehabilitation significantly improved physical and shoulder function indicators during the posttreatment and follow-up periods. For rotator cuff-related symptoms, various rehabilitation programs are available for selection. Ferlito et al^24^ demonstrated that limited exercise of the scapulothoracic complex during shoulder girdle movement is an effective alternative treatment for patients with subacromial impingement syndrome. Karamanlioglu et al^25^ reported that for patients with subacromial impingement syndrome, acupuncture treatment can relieve pain and improve shoulder function. However, previous rehabilitation programs usually placed patients in a passive role. By enhancing the accessibility of guideline-recommended care through telemedicine and achieving internet-based patient-oriented care, it is possible to improve healthcare quality and outcomes. One possible explanation for the improvement in the telemedicine group is that they received a broader, more frequent, and more continuous course of physical therapy.^26^ When patients in the telemedicine group were discharged from the hospital, the rehabilitation process did not break off, and the shoulder joint exercise training did not stop. Another possible explanation is that patients could stay at home to better prepare and focus on training.^18^ In general, patients are more likely to receive care from the physical therapist of their choice in the environment they have selected, which is crucial for the quality of their recovery. This method enhances the continuity of care and ensures patients receive necessary assistance and support when needed.^27^^,^^28^ Failure to carry out effective rehabilitation exercises can lead to joint adhesions, pain, and even the risk of a second surgery.^29^ The telemedicine team operated under the guidance of hospital physical therapists, providing patients with a more convenient and professional way to improve their function.

### Strengths and limitations of this study

#### Strengths

This study is the first to systematically demonstrate the clinical value of telemedicine in improving functional outcomes for patients after arthroscopic rotator cuff repair, providing evidence-based support for digital healthcare in orthopedic rehabilitation. The research results support the integration of remote intervention into a stepwise treatment plan, which has practical significance for optimizing medical resource allocation and improving the efficiency of chronic disease management.

#### Limitations

First, although a strong meta-analysis typically requires >9 studies, our analysis included only 5, which may limit the robustness of the findings. The comprehensiveness of our results may also be affected by insufficient data in some studies and unpublished records. Second, due to the limited number of studies, we could not fully estimate the diversity of telemedicine models or conduct subgroup analyses based on key demographic and clinical factors. Third, details regarding the frequency and intensity of both remote and conventional interventions were often poorly described across the included studies. Fourth, although 1 study suggested telemedicine offers social and economic benefits,^19^ we could not quantitatively assess cost-effectiveness, patient satisfaction, or treatment burden due to a lack of reported data. Given these limitations, more high-quality RCTs are urgently needed to confirm the effectiveness and safety of telemedicine, explore its applicability across different patient subgroups, and establish standardized protocols. Future research should also prioritize close monitoring and reporting of intervention compliance, as this significantly impacts the effectiveness of telemedicine.^30^

## Conclusion

In conclusion, this meta-analysis supports the claim that telemedicine is superior to traditional therapies in improving shoulder function following arthroscopic rotator cuff repair. The application of telemedicine in this patient population is an emerging and interesting field. However, further research is needed to fully understand its overall effectiveness, especially considering various surgical techniques and patient demographic characteristics.

## Suppliers

a. Cochrane risk of bias tool (ROB2).
b. RevMan, version 5.4.

## Disclosure

The authors declare that they have no known competing financial interests or personal relationships that could have appeared to influence the work reported in this paper.

## Appendix Supplementary materials

Image, application 1

Image, application 2

## Footnotes

Supplementary material associated with this article can be found, in the online version, at doi:10.1016/j.arrct.2026.100635.
