# Supplementary material for: The Effect of Telerehabilitation on the Outcome of Arthroscopic Rotator Cuff Repair: A Meta-analysis of Randomized Clinical Trials
Source: Arch Rehabil Res Clin Transl. 2026 May 13;8(3):100635. doi: 10.1016/j.arrct.2026.100635 (PMC13591186; doi:10.1016/j.arrct.2026.100635)
Supplement: Supplementary file 2 [file mmc2.docx]

**Table S2.** Search strategy

1. PubMed

| Search number | Query | Sort By | Filters | Search Details | Results | Time |
| --- | --- | --- | --- | --- | --- | --- |
| 1 | Telerehabilitation[MeSH Terms] | | | "telerehabilitation"[MeSH Terms] | 1,117 | 9:21:15 |
| 2 | "Telerehabilitation"[Title/Abstract] OR "Telerehabilitations"[Title/Abstract] OR "Tele-rehabilitation"[Title/Abstract] OR "Tele rehabilitation"[Title/Abstract] OR "Tele-rehabilitations"[Title/Abstract] OR "Remote Rehabilitation"[Title/Abstract] OR "Remote Rehabilitations"[Title/Abstract] OR "Virtual Rehabilitation"[Title/Abstract] OR "Virtual Rehabilitations"[Title/Abstract] OR "e-rehabilitation"[Title/Abstract] OR "remote rehabilitation"[Title/Abstract] OR "tele-rehabilitation"[Title/Abstract] OR "virtual rehabilitation"[Title/Abstract] | | | "Telerehabilitation"[Title/Abstract] OR "tele rehabilitation"[Title/Abstract] OR "tele rehabilitation"[Title/Abstract] OR "Remote Rehabilitation"[Title/Abstract] OR "Virtual Rehabilitation"[Title/Abstract] OR "Virtual Rehabilitations"[Title/Abstract] OR "e-rehabilitation"[Title/Abstract] OR "Remote Rehabilitation"[Title/Abstract] OR "tele rehabilitation"[Title/Abstract] OR "Virtual Rehabilitation"[Title/Abstract] | 2355 | 9:22:16 |
| 3 | Telemedicine[MeSH Terms] | | | "telemedicine"[MeSH Terms] | 36,178 | 9:23:23 |
| 4 | "Telemedicine"[Title/Abstract] OR "Tele-Referral"[Title/Abstract] OR "Tele Referral"[Title/Abstract] OR "Tele-Referrals"[Title/Abstract] OR "Virtual Medicine"[Title/Abstract] OR "Tele-Intensive Care"[Title/Abstract] OR "Tele Intensive Care"[Title/Abstract] OR "Tele-ICU"[Title/Abstract] OR "Tele ICU"[Title/Abstract] OR "Mobile Health"[Title/Abstract] OR "mHealth"[Title/Abstract] OR "Telehealth"[Title/Abstract] OR "eHealth"[Title/Abstract] OR "tele medicine"[Title/Abstract] OR "virtual medicine"[Title/Abstract] | | | "Telemedicine"[Title/Abstract] OR "tele referral"[Title/Abstract] OR "tele referral"[Title/Abstract] OR "Tele-Referrals"[Title/Abstract] OR "Virtual Medicine"[Title/Abstract] OR "tele intensive care"[Title/Abstract] OR "tele intensive care"[Title/Abstract] OR "tele icu"[Title/Abstract] OR "tele icu"[Title/Abstract] OR "Mobile Health"[Title/Abstract] OR "mHealth"[Title/Abstract] OR "Telehealth"[Title/Abstract] OR "eHealth"[Title/Abstract] OR "tele medicine"[Title/Abstract] OR "Virtual Medicine"[Title/Abstract] | 59,744 | 9:24:18 |
| 5 | #1 or #2 or #3 or #4 | |  | "Telerehabilitation"[MeSH Terms] OR "Telerehabilitation"[Title/Abstract] OR "tele rehabilitation"[Title/Abstract] OR "tele rehabilitation"[Title/Abstract] OR "Remote Rehabilitation"[Title/Abstract] OR "Virtual Rehabilitation"[Title/Abstract] OR "Virtual Rehabilitations"[Title/Abstract] OR "e-rehabilitation"[Title/Abstract] OR "Remote Rehabilitation"[Title/Abstract] OR "tele rehabilitation"[Title/Abstract] OR "Virtual Rehabilitation"[Title/Abstract] OR "Telemedicine"[MeSH Terms] OR "Telemedicine"[Title/Abstract] OR "tele referral"[Title/Abstract] OR "tele referral"[Title/Abstract] OR "Tele-Referrals"[Title/Abstract] OR "Virtual Medicine"[Title/Abstract] OR "tele intensive care"[Title/Abstract] OR "tele intensive care"[Title/Abstract] OR "tele icu"[Title/Abstract] OR "tele icu"[Title/Abstract] OR "Mobile Health"[Title/Abstract] OR "mHealth"[Title/Abstract] OR "Telehealth"[Title/Abstract] OR "eHealth"[Title/Abstract] OR "tele medicine"[Title/Abstract] OR "Virtual Medicine"[Title/Abstract] | 83,027 | 9:25:16 |
| 6 | Shoulder Joint[MeSH Terms] | | | "shoulder joint"[MeSH Terms] | 19,914 | 9:26:05 |
| 7 | "Shoulder Joint"[Title/Abstract] OR "Shoulder Joints"[Title/Abstract] OR "Glenohumeral Joint"[Title/Abstract] OR "Glenohumeral Joints"[Title/Abstract] OR "Glenoid Labrum"[Title/Abstract] OR "articulatio glenohumeralis"[Title/Abstract] OR "articulatio humeri"[Title/Abstract] OR "gleno-humeral joint"[Title/Abstract] OR "glenohumeral joint"[Title/Abstract] OR "humeral joint"[Title/Abstract] OR "humero-scapular joint"[Title/Abstract] OR "humeroscapular joint"[Title/Abstract] OR "scapulo humeral joint"[Title/Abstract] OR "scapulohumeral joint"[Title/Abstract] | | | "Shoulder Joint"[Title/Abstract] OR "Shoulder Joints"[Title/Abstract] OR "Glenohumeral Joint"[Title/Abstract] OR "Glenohumeral Joints"[Title/Abstract] OR "Glenoid Labrum"[Title/Abstract] OR "articulatio humeri"[Title/Abstract] OR "gleno-humeral joint"[Title/Abstract] OR "Glenohumeral Joint"[Title/Abstract] OR "humeral joint"[Title/Abstract] OR "humero-scapular joint"[Title/Abstract] OR "humeroscapular joint"[Title/Abstract] OR "scapulo humeral joint"[Title/Abstract] OR "scapulohumeral joint"[Title/Abstract] | 10,161 | 9:25:53 |
| 8 | Arthroplasty[MeSH Terms] | | | "arthroplasty"[MeSH Terms] | 13,525 | 9:26:34 |
| 9 | "Arthroplasty"[Title/Abstract] OR "Arthroplasties"[Title/Abstract] OR "Bone Tunnel Enlargement"[Title/Abstract] OR "Bone Tunnel Enlargements"[Title/Abstract] OR "Bone Tunnel Widening"[Title/Abstract] OR "Bone Tunnel Widenings"[Title/Abstract] OR "Alloarthroplasty"[Title/Abstract] OR "Arthroplasties"[Title/Abstract] OR "joint reconstruction"[Title/Abstract] OR "joint reconstructive procedure"[Title/Abstract] OR "joint reconstructive surgery"[Title/Abstract] | | | "Arthroplasty"[Title/Abstract] OR "Arthroplasties"[Title/Abstract] OR "Bone Tunnel Enlargement"[Title/Abstract] OR "Bone Tunnel Enlargements"[Title/Abstract] OR "Bone Tunnel Widening"[Title/Abstract] OR "Alloarthroplasty"[Title/Abstract] OR "Arthroplasties"[Title/Abstract] OR "joint reconstruction"[Title/Abstract] OR "joint reconstructive surgery"[Title/Abstract] | 64,312 | 9:27:30 |
| 10 | Arthroscopy[MeSH Terms] | | | "arthroscopy"[MeSH Terms] | 19,389 | 9:28:50 |
| 11 | "Arthroscopy"[Title/Abstract] OR "Arthroscopies"[Title/Abstract] OR "Arthroscopic Surgical Procedures"[Title/Abstract] OR "Arthroscopic Surgical Procedure"[Title/Abstract] OR "Arthroscopic Surgery"[Title/Abstract] OR "Arthroscopic Surgeries"[Title/Abstract] OR "Arthroendoscopy"[Title/Abstract] OR "arthroscopic exam"[Title/Abstract] OR "arthroscopic examination"[Title/Abstract] OR "arthroscopic procedure"[Title/Abstract] OR "arthroscopic procedures"[Title/Abstract] | | | "Arthroscopy"[Title/Abstract] OR "Arthroscopies"[Title/Abstract] OR "Arthroscopic Surgical Procedures"[Title/Abstract] OR "Arthroscopic Surgical Procedure"[Title/Abstract] OR "Arthroscopic Surgery"[Title/Abstract] OR "Arthroscopic Surgeries"[Title/Abstract] OR "Arthroendoscopy"[Title/Abstract] OR "arthroscopic exam"[Title/Abstract] OR "arthroscopic examination"[Title/Abstract] OR "arthroscopic procedure"[Title/Abstract] OR "arthroscopic procedures"[Title/Abstract] | 16,925 | 9:29:21 |
| 12 | "Rotator Cuff"[Mesh] | | | "Rotator Cuff"[Mesh] | 8,786 |  |
| 13 | (((((Rotator Cuff[Title/Abstract]) OR (Rotator Cuffs[Title/Abstract])) OR (Supraspinatus[Title/Abstract])) OR (Infraspinatus[Title/Abstract])) OR (Subscapularis[Title/Abstract])) OR (Teres Minor[Title/Abstract]) | | | (((((Rotator Cuff[Title/Abstract]) OR (Rotator Cuffs[Title/Abstract])) OR (Supraspinatus[Title/Abstract])) OR (Infraspinatus[Title/Abstract])) OR (Subscapularis[Title/Abstract])) OR (Teres Minor[Title/Abstract]) | 12,571 |  |
| 14 | #6 or #7 or #8 or #9 or #10 or #11 or #12 or #13 | | | ((((((("Shoulder Joint"[Mesh]) OR ("Shoulder Joint"[Title/Abstract] OR "Shoulder Joints"[Title/Abstract] OR "Glenohumeral Joint"[Title/Abstract] OR "Glenohumeral Joints"[Title/Abstract] OR "Glenoid Labrum"[Title/Abstract] OR "articulatio glenohumeralis"[Title/Abstract] OR "articulatio humeri"[Title/Abstract] OR "gleno-humeral joint"[Title/Abstract] OR "glenohumeral joint"[Title/Abstract] OR "humeral joint"[Title/Abstract] OR "humero-scapular joint"[Title/Abstract] OR "humeroscapular joint"[Title/Abstract] OR "scapulo humeral joint"[Title/Abstract] OR "scapulohumeral joint"[Title/Abstract])) OR ("Arthroplasty"[Mesh])) OR ("Arthroplasty"[Title/Abstract] OR "Arthroplasties"[Title/Abstract] OR "Bone Tunnel Enlargement"[Title/Abstract] OR "Bone Tunnel Enlargements"[Title/Abstract] OR "Bone Tunnel Widening"[Title/Abstract] OR "Bone Tunnel Widenings"[Title/Abstract] OR "Alloarthroplasty"[Title/Abstract] OR "Arthroplasties"[Title/Abstract] OR "joint reconstruction"[Title/Abstract] OR "joint reconstructive procedure"[Title/Abstract] OR "joint reconstructive surgery"[Title/Abstract])) OR ("Rotator Cuff"[Mesh])) OR ((((((Rotator Cuff[Title/Abstract]) OR (Rotator Cuffs[Title/Abstract])) OR (Supraspinatus[Title/Abstract])) OR (Infraspinatus[Title/Abstract])) OR (Subscapularis[Title/Abstract])) OR (Teres Minor[Title/Abstract]))) OR ("Arthroscopy"[Mesh])) OR ("Arthroscopy"[Title/Abstract] OR "Arthroscopies"[Title/Abstract] OR "Arthroscopic Surgical Procedures"[Title/Abstract] OR "Arthroscopic Surgical Procedure"[Title/Abstract] OR "Arthroscopic Surgery"[Title/Abstract] OR "Arthroscopic Surgeries"[Title/Abstract] OR "Arthroendoscopy"[Title/Abstract] OR "arthroscopic exam"[Title/Abstract] OR "arthroscopic examination"[Title/Abstract] OR "arthroscopic procedure"[Title/Abstract] OR "arthroscopic procedures"[Title/Abstract]) | 190,178 | 9:30:16 |
| 15 | #5 and #14 | |  | (((((((("Shoulder Joint"[Mesh]) OR ("Shoulder Joint"[Title/Abstract] OR "Shoulder Joints"[Title/Abstract] OR "Glenohumeral Joint"[Title/Abstract] OR "Glenohumeral Joints"[Title/Abstract] OR "Glenoid Labrum"[Title/Abstract] OR "articulatio glenohumeralis"[Title/Abstract] OR "articulatio humeri"[Title/Abstract] OR "gleno-humeral joint"[Title/Abstract] OR "glenohumeral joint"[Title/Abstract] OR "humeral joint"[Title/Abstract] OR "humero-scapular joint"[Title/Abstract] OR "humeroscapular joint"[Title/Abstract] OR "scapulo humeral joint"[Title/Abstract] OR "scapulohumeral joint"[Title/Abstract])) OR ("Arthroplasty"[Mesh])) OR ("Arthroplasty"[Title/Abstract] OR "Arthroplasties"[Title/Abstract] OR "Bone Tunnel Enlargement"[Title/Abstract] OR "Bone Tunnel Enlargements"[Title/Abstract] OR "Bone Tunnel Widening"[Title/Abstract] OR "Bone Tunnel Widenings"[Title/Abstract] OR "Alloarthroplasty"[Title/Abstract] OR "Arthroplasties"[Title/Abstract] OR "joint reconstruction"[Title/Abstract] OR "joint reconstructive procedure"[Title/Abstract] OR "joint reconstructive surgery"[Title/Abstract])) OR ("Rotator Cuff"[Mesh])) OR ((((((Rotator Cuff[Title/Abstract]) OR (Rotator Cuffs[Title/Abstract])) OR (Supraspinatus[Title/Abstract])) OR (Infraspinatus[Title/Abstract])) OR (Subscapularis[Title/Abstract])) OR (Teres Minor[Title/Abstract]))) OR ("Arthroscopy"[Mesh])) OR ("Arthroscopy"[Title/Abstract] OR "Arthroscopies"[Title/Abstract] OR "Arthroscopic Surgical Procedures"[Title/Abstract] OR "Arthroscopic Surgical Procedure"[Title/Abstract] OR "Arthroscopic Surgery"[Title/Abstract] OR "Arthroscopic Surgeries"[Title/Abstract] OR "Arthroendoscopy"[Title/Abstract] OR "arthroscopic exam"[Title/Abstract] OR "arthroscopic examination"[Title/Abstract] OR "arthroscopic procedure"[Title/Abstract] OR "arthroscopic procedures"[Title/Abstract])) AND (((("Telerehabilitation"[Mesh]) OR ("Telerehabilitation"[Title/Abstract] OR "Telerehabilitations"[Title/Abstract] OR "Tele-rehabilitation"[Title/Abstract] OR "Tele rehabilitation"[Title/Abstract] OR "Tele-rehabilitations"[Title/Abstract] OR "Remote Rehabilitation"[Title/Abstract] OR "Remote Rehabilitations"[Title/Abstract] OR "Virtual Rehabilitation"[Title/Abstract] OR "Virtual Rehabilitations"[Title/Abstract] OR "e-rehabilitation"[Title/Abstract] OR "remote rehabilitation"[Title/Abstract] OR "tele-rehabilitation"[Title/Abstract] OR "virtual rehabilitation"[Title/Abstract])) OR ("Telemedicine"[Mesh])) OR ("Telemedicine"[Title/Abstract] OR "Tele-Referral"[Title/Abstract] OR "Tele Referral"[Title/Abstract] OR "Tele-Referrals"[Title/Abstract] OR "Virtual Medicine"[Title/Abstract] OR "Tele-Intensive Care"[Title/Abstract] OR "Tele Intensive Care"[Title/Abstract] OR "Tele-ICU"[Title/Abstract] OR "Tele ICU"[Title/Abstract] OR "Mobile Health"[Title/Abstract] OR "mHealth"[Title/Abstract] OR "Telehealth"[Title/Abstract] OR "eHealth"[Title/Abstract] OR "tele medicine"[Title/Abstract] OR "virtual medicine"[Title/Abstract])) | 287 | 9:31:32 |
